# Phylogenetic Analysis of Different Ploidy *Saccharum spontaneum* Based on rDNA-ITS Sequences

**DOI:** 10.1371/journal.pone.0151524

**Published:** 2016-03-17

**Authors:** Xinlong Liu, Xujuan Li, Hongbo Liu, Chaohua Xu, Xiuqin Lin, Chunjia Li, Zuhu Deng

**Affiliations:** 1 Key Lab of Sugarcane Biology and Genetic Breeding, Ministry of Agriculture, Fujian Agriculture and Forestry University, Fuzhou, China; 2 Yunnan Key Laboratory of Sugarcane Genetic Improvement, Sugarcane Research Institute, Yunnan Academy of Agricultural Sciences, Kaiyuan, China; National Cheng-Kung University, TAIWAN

## Abstract

*Saccharum spontaneum* L. is a crucial wild parent of modern sugarcane cultivars whose ploidy clones have been utilized successfully in improving the stress resistance and yield related traits of sugarcane cultivars. To establish knowledge regarding the genetic variances and evolutional relationships of ploidy clones of *Saccharum spontaneum* collected in China, the rDNA-ITS sequences of 62 ploidy clones including octaploid clones (2n = 64), nonaploid clones (2n = 72), decaploid clones (2n = 80), and dodecaploid clones (2n = 96), were obtained and analyzed. The rDNA-ITS sequences of four species from *Saccharum* and *Sorghum bicolor* selected as controls. The results showed that decaploid clones (2n = 80) possess the most abundant variances with 58 variable sites and 20 parsim-informative sites in ITS sequences, which were then followed by octaploid clones with 43 variable sites and 17 parsim-informative sites. In haplotype diversity, all four population exhibited high diversity, especially nonaploid and decaploid populations. By comparing the genetic distances among four ploidy populations, the dodecaploid population exhibited the closest relationship with the nonaploid population, and then the relationship strength decreased successively for the decaploid population and then for the octaploid population. Population differentiation analysis showed that the phenomena of population differentiation were not found among different ploidy populations, and low coefficient of gene differentiation(Gst) and high gene flow(Nm) occur among these populations possessing close genetic relationship. These results mentioned above will contribute to the understanding of the evolution of different ploidy populations of *Saccharum spontaneum* and provide vital knowledge for their utilization in sugarcane breeding and innovation.

## Introduction

*Saccharum spontaneum* L. is a crucial wild parent for modern sugarcane cultivars that can improve cultivars with regards to tolerances to abiotic or biotic stress and yield related traits. More notably it has the widest ecogeographical distribution among *Saccharum spp*. and different clones show wide morphological variation [1]. Clones vary from short bushy types with reduced leaves to midrib and practically no formation to tall, erect, broad-leaved forms with long internodes [2]. In China, the plant is distributed mainly in the central, southern, and southwest parts [3].

*S*. *spontaneum* also belongs to the complex polyploid plants like sugarcane, whose chromosome number has been reported to range from 2n = 40 to 128 with basic number of chromosome x = 8, and five types of chromosome number (2n = 64, 80, 96, 112, and 128) appear to be distributed highly frequency [4–6]. Studies using GISH and FISH techniques dissected the chromosome composition of model sugarcane cultivars revealed that approximately 10–20% of total chromosomes of cultivars come from *S*. *spontaneum* and that about 10% of these occurred in the inter-specific exchange between *S*. *spontaneum* and *S*. *officinarum* [7–9]. These studies have confirmed that *S*. *spontaneum* has become an important component of modern sugarcane cultivars.

During the past several years, research communities around the world have mainly focused their studies on the genetic diversity of clones of *S*. *spontaneum* collected from different areas. This studies have proved that *S*. *spontaneum* possess very rich genetic variances in phenotypic and molecular level traits [10–15]. In addition, because *S*. *spontaneum* is easy to cross with *S*. *officinarum* & sugarcane cultivars and their offspring exhibit good performance in stress resistance, adaptability, and ratoon capability *S*. *spontaneum* is regarded as one of the most valuable wild specie for exploring sugarcane breeding [1].

When reviewing the history of sugarcane breeding, it is unfortunate that only limited euploid clones of *S*. *spontaneum*, including Glagah (2n = 112), Indian (2n = 64), and Yacheng (2n = 64, 80), have been utilized successfully in sugarcane breeding and were used to make series of elite parents such as the POJ series, the Co series and the Yacheng series. To date, these parents still play a critical role in breeding [1, 16].

Since the 1970s, the collecting work of *S*. *spontaneum* has been carried out in China. At present about 700 clones, which were collected from Yunnan, Guangxi, Guangdong, Fujian, Sichuan, and Hainan, were conserved in the China National Nursery of Sugarcane Germplasm Resources (CNNSGR) in Kaiyuan, Yunnan province. By identifying the chromosome number of 247 clones conserved in CNNSGR, Cai et al. [17] found 11 chromosome types that are 2n = 60, 64, 70, 72, 74, 78, 80, 92, 96, 104, 108 and 4 which were euploid types (2n = 64, 72, 80, 96) make up a high percentage in all identified clones. Currently, the genetic background and evolutionary relationships for these euploid clones still remain unclear, which has limited their utilization in sugarcane breeding.

At present, some sequences such as rDNA-ITS, rbcl, apha-tubulin, rpl16 and rpoC2 have been using in the genetic relationship analysis of these species belong to *Saccharum* L. and other related genus as *Miscanthus* Anderss., *Erianthus* Michaux and *Narenga* Bor. [18–23]. These previous studies demonstrated that these sequences except rDNA-ITS are very conserved among different species, which suit for evolutionary analysis of different genus. For rDNA-ITS, the characters of rich variances, rapid evolutionary rate and easy PCR amplification make it a very important marker used in the evolutionary analysis of "sugarcane complex", and it also is often used to evaluate evolutionary relationships at the subspecies level [24]. In view of this, 62 ploidy clones belonging to 4 euploid types of *S*. *spontaneum*, were evaluated in this study for genetic variances and phylogenetic relationships via the rDNA-ITS sequences. The results will provide informative knowledge for utilization in sugarcane breeding and innovation.

## Materials and Methods

### Ethics Statement

*S*. *spontaneum* is not considered an endangered species, collecting is allowed in field environment. These *S*. *spontaneum* clones in this study were collected in recent decades by Yunnan Sugarcane Research Institute (YSRI). At present, these clones were conserved in the CNNSGR (China National Nursery of Sugarcane Germplasm Resources), which was built by China's Ministry of Agriculture in Kaiyuan city, Yunan province in 1995. The YSRI was entrusted with managing the routine works of CNNSGR. We were assigned to responsible for management, evaluation of these resources by YSRI. Finally, we confirm that no specific permits were required for the present studies.

### Plant materials

A total of 62 different ploidy clones of *S*. *spontaneum* were selected from CNNSGR, of which 45 clones (23 decaploid clones and 22 octaploid clones) were chosen according to the standard of one clone per county with reference to their collection location. Because there were only 7 nonaploid clones and 10 dodecaploid clones conserved in CNNSGR, all of these clones were chosen for this study. And 31 rDNA-ITS sequences from five species (*Saccharum officinarum*, *Saccharum barberi*, *Saccharum sinense*, *Saccharum robustum* and *Sorghum bicolor*) downloaded from GenBank were regarded as controls. All clones and control sequences were listed in Tables 1 and 2 in detail.

**Table 1:** The list of clones of *S*. *spontaneum* used in this study.

No.	Sample name	Ploidy type/Chromosome number	Collected location	GenBank accession No.
1	Yunnan82-59	Octaploid/2n = 64	Binchuan county, Yunnan	KJ934283
2	Yunnan82-149	Octaploid/2n = 64	Changning county, Yunnan	KJ934287
3	Yunnan83-238	Octaploid/2n = 64	Dayao county, Yunnan	KJ934293
4	Yunnan75-2-2	Octaploid/2n = 64	Eshan county, Yunnan	KJ934276
5	Yunnan82-79	Octaploid/2n = 64	Gengma county, Yunnan	KJ934285
6	Yunnan83-160	Octaploid/2n = 64	Hekou county, Yunnan	KJ934288
7	Yunnan4	Octaploid/2n = 64	Honghe county, Yunnan	KJ934274
8	Yunnan82-20	Octaploid/2n = 64	Lianghe county, Yunnan	KJ934280
9	Yunnan83-227	Octaploid/2n = 64	Liuku county, Yunnan	KJ934291
10	Yunnan83-225	Octaploid/2n = 64	Lushui county, Yunnan	KJ934290
11	Yunnan75-1-10	Octaploid/2n = 64	Mang city, Yunnan	KJ934275
12	Yunnan84-268	Octaploid/2n = 64	Mang city, Yunnan	KJ934294
13	Yunnan Mengzi	Octaploid/2n = 64	Mengzi county, Yunnan	KJ934273
14	Yunnan82-63	Octaploid/2n = 64	Nanjian county, Yunnan	KJ934284
15	Yunnan82-9	Octaploid/2n = 64	Ruili city, Yunnan	KJ934278
16	Yunnan82-25	Octaploid/2n = 64	Tengchong county, Yunnan	KJ934281
17	Yunnan83-213	Octaploid/2n = 64	Yangbi county, Yunnan	KJ934289
18	Yunnan82-14	Octaploid/2n = 64	Yingjiang county, Yunnan	KJ934279
19	Yunnan83-228	Octaploid/2n = 64	Yongping county, Yunnan	KJ934292
20	Yunnan82-58	Octaploid/2n = 64	Rongsheng county, Yunnan	KJ934282
21	Yunnan75-2-11	Octaploid/2n = 64	Yuanjiang county, Yunnan	KJ934277
22	Yunnan82-140	Octaploid/2n = 64	Yuanyang county, Yunnan	KJ934286
23	Fujian89-1-11	Nonaploid/2n = 72	Gutian county, Fujian	KJ934297
24	Fujian89-1-1	Nonaploid/2n = 72	Songxi county, Fujian	KJ934296
25	Guizhou78-1-11	Nonaploid/2n = 72	Xishui county, Guizhou	KJ934298
26	Yunnan76-1-016	Nonaploid/2n = 72	Miyi county, Sichuan	KJ934300
27	Sichuan92-42	Nonaploid/2n = 72	Leshan city, Sichuan	KJ934299
28	Yunnan82-50	Nonaploid/2n = 72	Huaping county, Yunnan	KJ934295
29	Yunnan83-201	Nonaploid/2n = 72	Yanjing county, Yunnan	KJ934301
30	Fujian Dongshan	Decaploid/2n = 80	Dongshan county, Fujian	KJ934334
31	Fujian92-1-11	Decaploid/2n = 80	Fuzhou city, Fujian	KJ934333
32	Fujian87-1-14	Decaploid/2n = 80	Lizhi,Putian city, Fujian	KJ934332
33	Fujian89-1-21	Decaploid/2n = 80	Xiamen city, Fujian	KJ934303
34	Guangdong16	Decaploid/2n = 80	Guangzhou city, Guangdong	KJ934304
35	Guangdong35	Decaploid/2n = 80	Huazhou city, Guangdong	KJ934307
36	Guangdong31	Decaploid/2n = 80	Luhe county, Guangdong	KJ934306
37	Guangdong Shaoguan	Decaploid/2n = 80	Ruiyuan county, Guangdong	KJ934311
38	Guizhou78-2-4	Decaploid/2n = 80	Rongjiang county, Guizhou	KJ934312
39	Guizhou78-1-31	Decaploid/2n = 80	Sinan county, Guizhou	KJ934338
40	Guizhou78-1-5	Decaploid/2n = 80	Xishui county, Guizhou	KJ934337
41	Guizhou84-260	Decaploid/2n = 80	Xingyi city, Guizhou	KJ934302
42	Hainan Ledong1	Decaploid/2n = 80	Huangliu county, Hainan	KJ934340
43	Sichuan79-1-26	Decaploid/2n = 80	DA county, Sichuan	KJ934313
44	Sichuan88-41	Decaploid/2n = 80	Jitang county, Sichuan	KJ934343
45	Sichuan79-2-1	Decaploid/2n = 80	Lushui county, Sichuan	KJ934341
46	Yunnan75-2-35	Decaploid/2n = 80	Hekou county, Yunnan	KJ934346
47	Yunnan76-3-2	Decaploid/2n = 80	Jinghong city, Yunnan	KJ934324
48	Yunnan82-12	Decaploid/2n = 80	Longchuan county, Yunnan	KJ934325
49	Yunnan82-44	Decaploid/2n = 80	Zhongdian county, Yunnan	KJ934326
50	Chongqing76-1-024	Decaploid/2n = 80	Miyi county, Chongqing	KJ934347
51	Chongqing79-2-13	Decaploid/2n = 80	Wanzhou district, Chongqing	KJ934342
52	Chongqing79-2-16	Decaploid/2n = 80	Yunyang county, Chongqing	KJ934316
53	Fujian Huian	Dodecaploid/2n = 96	Huian county, Fujian	KJ934358
54	Fujian88-1-12	Dodecaploid/2n = 96	Nanjing county, Fujian	KJ934353
55	Fujian88-1-13	Dodecaploid/2n = 96	Nanjing county, Fujian	KJ934354
56	Fujian89-1-16	Dodecaploid/2n = 96	Putian city, Fujian	KJ934355
57	Fujian89-1-17	Dodecaploid/2n = 96	Putian city, Fujian	KJ934356
58	Fujian89-1-18	Dodecaploid/2n = 96	Putian city, Fujian	KJ934357
59	Fujian Xianyou	Dodecaploid/2n = 96	Putian city, Fujian	KJ934359
60	Guangdong30	Dodecaploid/2n = 96	Haifeng county, Guangdong	KJ934360
61	Guizhou78-2-28	Dodecaploid/2n = 96	Sanjiang county, Guizhou	KJ934361
62	Sichuan79-2-11	Dodecaploid/2n = 96	Zhong county, Chongqing	KJ934352

**Table 2:** The list of control rDNA-ITS sequences.

Specie name	Sample name	GenBank accession No.
*Saccharum officinarum*	Mangeer, Orambo, genotype104, R3, R1, R2, Skendzic5068, Karia	AB250692.1, AB250691.1, AF345231.1, AF345229.1, AF345230.1, DQ005064.1, AB250693.1
*Saccharum barberi*	Nargori, PutjeeKhajee, Dhaurkinara, R5, R4, R6	AB281150.1, AB281148.1, AB281149.1, AF345199.1, AF331657.1, AF345200.1
*Saccharum sinense*	Khelia, Tukya1, Khakai, R8, R10, R9, R7	AB281153.1, AB281154.1, AB281152.1, AF345242.1, AF345240.1, AF345243.1, AF345241.1
*Saccharum robustum*	NG-77-27, R12, R13, R11	AB281156.1, AF345238.1, AF345239.1, AF345237.1
*Sorghum bicolor*	Vu12, Vu11, B1, B2, B3, B4	DQ190421.1, DQ190420.1, GQ856358.1, GQ121748.1, GQ121745.1, GQ121744.1, GQ121743.1

### DNA extraction and PCR amplification

Considering all stalks per clone arise from these rhizome buds through vegetative propagation, the mixed young tender leaves from multiple stalks per clone were powdered with liquid nitrogen, then the genomic DNA of which was extracted by using the traditional CTAB method, the quality and concentration of DNA were respectively tested with 0.8% agarose gel and Thermo Nanodrop 2000, and then obtained DNA samples were diluted to the concentration of 20 ng/μl with deionized water for PCR amplification.

The rDNA-ITS region of all samples, which contain ITS1, 5.8s, and ITS2 regions, were amplified through using the universal primers ITS4 and ITS5(ITS4 primer sequence: 5’-TCCTCCGCTTATTGATATGC-3’, ITS5 primer sequence: 5’-GGAAGTAAAAGTCGTAACAAGG-3’) [25]. In view of lots of clones and shorter amplification sequence length, the High Fidelity TransTaq DNA Polymerase from Transgen biotech company, whose fidelity of PCR amplification is 18 times than common Taq polymerase, was employed for amplifying these short sequences instead of using PCR replication experiment to reduce the PCR amplification error. The PCR reaction system and procedures were performed according to Chen et al. [22]. PCR was performed on a Mastercycler gradient thermocycler (Eppendorf, Germany). The PCR products were tested by 1.0% agarose gel electrophoresis and then were purified using the OMEGAEZNA Gel extraction Kit. The purified PCR product was cloned into a PMD19-T vector, and the recombinant plasmids were transformed into a DH5α competent cell. In order to further increase the accuracy of sequence, five transformed clones per sample were selected for bi-directional sequencing by the BGI Company, China, then the sequence occupying the highest proportion among five sequences each sample was used for analysis. Finally, all obtained ITS sequences were uploaded to GenBank, the sequence accession No. per sample was list in Table 1.

### Sequence alignment and analyses

All obtained right sequences were aligned using the Clustal W program [26] with default settings. The basic sequence statistics including GC content, variable sites, and parsim-informative sites were counted through MEGA 6.06 software [27]. In view of DnaSP5.0 [28] and Arlequin3.11 [29] softwares successfully used to estimate nucleotide diversity of DNA or gene sequences and population differentiation of ployploid plants such as wheat [30–32] and potato [33,34], the two softwares were also used for rDNA-ITS sequence analysis of *S*. *spontaneum* clones. The haplotype diversity, nucleotide diversity, average number of nucleotide difference, gene flow(Nm) and coefficient of gene differentiation (Gst) were calculated according to these formulas (equation 8.4, equation 10.5 and equation 5) from Nei’s reports [35,36] by using DnaSP5.0 software; and the analysis of molecular variance among populations were implemented by using Arlequin 3.11 software to calculate the Variance of components, Percentage of variation, fixation Index according to the standard AMOVN computations method with choosing haplotypic data and DNA type as data parameter type.

The genetic distances among four different ploidy populations were calculated according to Kimura 2-Parameter model using MEGA6.06 software. Differences in genetic distance between intra-population and inter-population were assessed by using independent-samples T test at P<0.05. The maximum-likelihood (ML) and neighbor-joining (NJ) method were used to construct a haplotype phylogenetic tree according to the Kimura 2-Parameter model using MEGA6.06 software, and all branches were evaluated with 1000 bootstrap replications and the trees with bootstrap confidence values >50% appear in the phylogenetic tree.

## Results

### Component and variance analysis of ITS sequences

Regarding the length of ITS sequences, there was only a types of sequences length in ITS1 sequences (207 bp) and 5.8S rDNA sequences (164 bp), and three length types (218 bp, 219 bp, and 220 bp) in ITS2 sequences. With regards to GC content, the value of GC content in ITS2 sequences with a mean of 69.3% is higher than that in ITS1 sequences with a mean of 63.5% (Table 3). 5.8S rDNA sequences exhibited the lowest GC content with a mean of 57.1%. Among different ploidy populations, there are no significant differences found in GC content.

**Table 3:** The GC content analysis of composition of ITS sequence of different ploidy populations of *S*. *spontaneum*.

	ITS1 GC content (%)		5.8s rDNA GC content (%)		ITS2 GC content (%)	
Population	Range	Mean	Range	Mean	Range	Mean
Octaploid	61.8–64.7	63.7	56.1–57.3	57.2	68.5–69.7	69.4
Nonaploid	61.4–64.3	63.1	56.1–57.3	57.1	67.9–69.7	69.1
Decaploid	61.8–64.7	63.4	56.1–57.9	57.1	67.6–69.9	69.1
Dodecaploid	62.3–64.7	63.4	56.7–57.3	57.0	68.3–70.3	69.4
Mean		63.5		57.1		69.3

According to the results of ITS sequences aligned using the Clustal W program, every ploidy population had 207 sites found in ITS1 sequences. However, there were differences in the number of sites for ITS2 sequences among different ploidy populations with 222 in an octaploid population, 220 in a decaploid population, and 219 in nonaploid and dodecaploid populations. For ITS sequences variable sites, the decaploid population had more rich variable sites with total 58 variable sites and 20 parsim-informative sites (20 variable sites and 13 parsim-informative sites in ITS1 sequences, 11 variable sites and 1 parsim-informative sites in 5.8S rDNA sequences, 27 variable sites and 6 parsim-informative sites in ITS2 sequences), which made up 9.81% and 3.38% of total sites respectively (Table 4). The ranked second for variances of ITS sequences is the octaploid population with total 43 variable sites and 17 parsim-informative sites. Then the dodecaploid and nonaploid populations exhibited low number of variable sites. As mentioned above, the largest variances of ITS sequences arise in the decaploid population, followed by the octaploid population. This may be due to the number of clones selected in this study.

**Table 4:** The analysis of variable sites of ITS sequence of different ploidy populations of *S*. *spontaneum*.

Population	Site name	ITS1	5.8s rDNA	ITS2	Total	Percentage of total sites (%)
Octaploid	Variable sites	20	7	16	43	7.25
	Parsim-informative sites	9	1	7	17	2.87
Nonaploid	Variable sites	12	2	11	25	4.24
	Parsim-informative sites	8	1	4	13	2.20
Decaploid	Variable sites	20	11	27	58	9.81
	Parsim-informative sites	13	1	6	20	3.38
Dodecaploid	Variable sites	14	1	11	26	4.41
	Parsim-informative sites	7	1	3	11	1.86

### Haplotype diversity analysis of population

The results of haplotype diversity analysis among four populations showed that total 51 haplotypes were found in four ploidy populations (Table 5), there were 20 haplotypes in octaploid population, 7 in nonaploid population, 22 in decaploid population and 8 in dodecaploid population. Hap2 and Hap3 were shared by three populations; Hap4 and Hap18 were shared by two populations. In the aspect of haplotype diversity, all four populations exhibited high diversity, the haplotype diversity (*Hd*) value ranged from 0.9333 to 1.0000 (Table 5). Nonaploid population performed the highest diversity, followed by decaploid population. Similarly, the high diversity in nonaploid and decaploid populations was also found in nucleotide diversity (*Pi*) because of high *Pi* value (0.0174 and 0.0177). Moreover, the two populations also appear big nucleotide difference, varying from 10.1905–10.3795.

**Table 5:** Haplotype diversity, nucleotide diversity of different ploidy populations of *S*. *spontaneum* according to rDNA-ITS haplotype data.

Population	Haplotype	Haplotype diversity (*Hd*)	Nucleotide diversity (*Pi*)	Average number of Nucleotide difference (k)
Octaploid	Hap3,18,34–51	0.9870±0.077	0.0154	9.0260
Nonaploid	Hap2,3,29–33	1.0000±0.077	0.0174	10.1905
Decaploid	Hap1-22	0.9961±0.014	0.0177	10.3795
Dodecaploid	Hap2,4,23–28	0.9333±0.077	0.0141	8.2444

**Fig 1:**
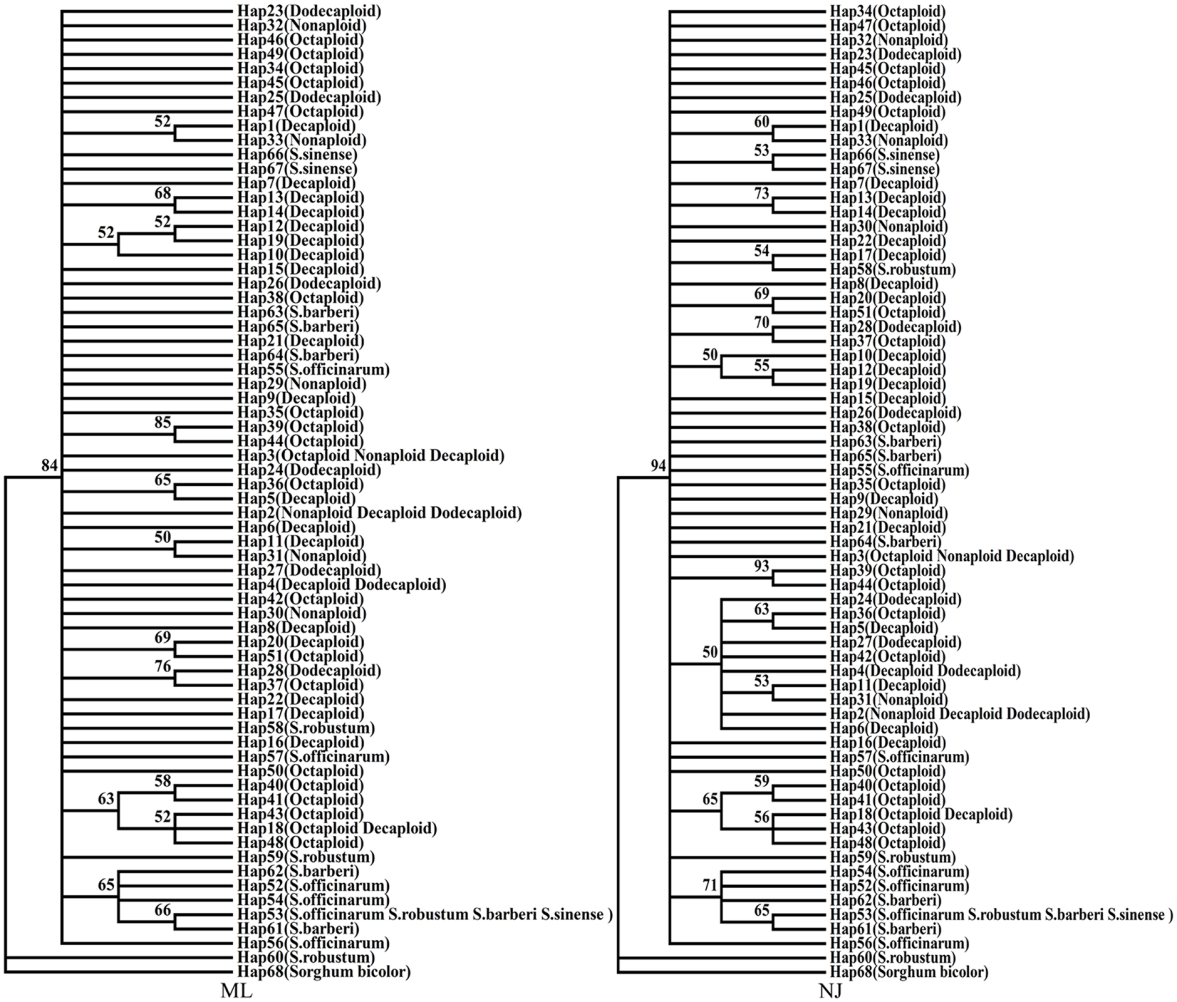
The ML and NJ phylogenetic tree based on rDNA-ITS haplotype data of different polyploid clones of *S*. *spontaneum*.

Using 17 haplotype data of rDNA-ITS sequence as outgroup, 16 of which from four species of *Saccharum (S*.*officinarum*, *S*.*robustum*, *S*.*barberi and S*.*sinense)* and 1 from *Sorghum bicolor*. Two phylogenetic trees with bootstrap confidence values >50% were constructed based on a Kimura 2-parameter model using the maximum-likelihood (ML) and neighbor-joining (NJ) methods (Fig 1). The results showed that the NJ tree was similar to the ML tree. For the two trees, the Hap68 from *Sorghum bicolor* and Hap60 from *S*.*robustum* separated firstly from the largest group consisting of 66 remained haplotypes. In the big group, 5 haplotypes from *S*.*officinarum*, *S*.*robustum*, *S*.*barberi and S*.*sinense* were clustered together with 71% and 65% bootstrap value in NJ and ML, and 5 haplotypes from octaploid and decaploid populations were assigned into another small group with 65% or 63% bootstrap value. Because the haplotypes from same population did not cluster together instead of exhibiting confused clustering relationships, these haplotypes from different ploidy populations were not obvious differentiation.

### Genetic distance among populations

By using a Kimura 2-parameter model of MEGA6.06 software, the mean genetic distances among different ploidy populations were obtained. The results are listed in Table 6. Four populations showed a close genetic relationship, of which nonaploid population and dodecaploid population exhibited the closest relationship with the smallest genetic distance of 0.0156. The genetic distances (0.0162) among dodecaploid population and decaploid or octaploid population were ranked as second. However, octaploid population and nonaploid displayed the farthest genetic relationship with the biggest genetic distance of 0.0171.

**Table 6:** The T test of genetic distance difference between inter-population and intra-population obtained using Kimura 2-parameter model.

Inter-population type	Mean pairwise distance among individuals of inter-population	Mean pairwise distance among individuals of intra-population	T test of pairwise distances between inter-population and intra-population
Octaploid and Nonaploid	0.0171(N = 154)	Octaploid: 0.0150(N = 231)	0.004*
		Nonaploid: 0.0177(N = 21)	0.737
Octaploid and Decaploid	0.0170(N = 506)	Octaploid: 0.0150(N = 231)	0.000*
		Decaploid: 0.0178(N = 253)	0.157
Octaploid and Dodecaploid	0.0163(N = 220)	Octaploid: 0.0150(N = 231)	0.029*
		Dodecaploid: 0.0143(N = 45)	0.127
Nonaploid and Decaploid	0.0170(N = 161)	Nonaploid: 0.0177(N = 21)	0.713
		Decaploid: 0.0178(N = 253)	0.122
Nonaploid and Dodecaploid	0.0156(N = 70)	Nonaploid: 0.0177(N = 21)	0.321
		Dodecaploid: 0.0143(N = 45)	0.396
Decaploid and Dodecaploid	0.0162(N = 230)	Decaploid: 0.0178(N = 253)	0.024*
		Dodecaploid: 0.0143(N = 45)	0.168

**Note**: N stands for pairwise distance number;

* indicates a statistically significant difference at p<0.05

In order to determine whether a reliable phylogenic tree of four populations can be constructed successfully according to ITS sequence data. The differences of genetic distance between inter-population and intra-population were assessed using independent-samples T test. The results exhibited that the genetic distances of inter-populations have no significant bigger than that of intra-population at P<0.05 (Table 6), which means that the reliability of population phylogenic tree may be interfered by intra-population variation. According to the situation above, a reliable phylogenic tree among four populations cannot be constructed.

### Population differentiation

The coefficient of gene differentiation (Gst), Gene flow and molecular variance were computed by using DnaSP5.0 and Arlequin 3.11 softwares. the results exhibited that the lowerest Gst value (0.0191), the highest Nm value (12.83) were obtained between nonaploid and decaploid populations (Table 7), this result indicated that two populations have high frequency gene exchanging, followed by the Gst (0.0314)and Nm (7.71) value between decaploid and dodecaploid populations. Between octaploid and dodecaploid populations, the biggest Gst value (0.0814) and the lowest Nm value (2.82) implied that low genetic exchanging occurred between two populations, similar result also appeared between octaploid and nonaploid populations. AMOVA analysis indicated that there was no significant differentiation among four ploidy populations at significance level of 0.001 with a low fixation index (0.0403) (Table 8). And the most of the variation (95.97%) was from within populations, only 4.03% variation from among populations. On comparison the percentage of variation of among population, the biggest value of 10.96% between octaploid and dodecaploid populations implied that there were more genetic differences between two populations, followed by between octaploid and nonaploid populations with a value of 8.26%. The results were consistent with the analysis of coefficient of gene differentiation (Gst) and Gene flow.

**Table 7:** Pairwise Gst (above the diagonal) and Nm (below the diagonal) among different ploidy populations according to rDNA-ITS data.

Population	Octaploid	Nonaploid	Decaploid	Dodecaploid
Octaploid		0.0621	0.0436	0.0814
Nonaploid	3.78		0.0191	0.0544
Decaploid	5.49	12.83		0.0314
Dodecaploid	2.82	4.35	7.71	

**Table 8:** Molecular variance (AMOVA) analysis among different ploidy populations according to rDNA-ITS haplotype data.

Group	Source of variation	df	Sum of squares	Variance of components	Percentage of variation (%)	Fixation index
Octaploid and Nonaploid	among populations	1	10.48	0.48	8.26	0.0826
	within populations	27	144.69	5.36	91.74	
	Total	28	155.17	5.84		
Octaploid and Decaploid	among populations	1	11.49	0.26	4.56	0.0456
	within populations	43	238.29	5.54	95.44	
	Total	44	249.78	5.81		
Octaploid and Dodecaploid	among populations	1	13.70	0.63	10.96	0.1096
	within populations	30	152.65	5.09	89.04	
	Total	31	166.34	5.71		
Nonaploid and Decaploid	among populations	1	3.22	-0.23	-4.24	-0.0424
	within populations	28	159.88	5.71	104.24	
	Total	29	163.10	5.48		
Nonaploid and Dodecaploid	among populations	1	4.52	-0.05	-1.06	-0.0106
	within populations	15	74.24	4.95	101.06	
	Total	16	78.77	4.90		
Decaploid and Dodecaploid	among populations	1	5.34	-0.01	-0.09	-0.0010
	within populations	31	167.84	5.41	100.09	
	Total	32	173.18	5.41		
Total	among populations	3	25.96	0.23	4.03	0.0403
	within populations	58	312.53	5.39	95.97	
	Total	61	338.48	5.61		

## Discussion

*S*. *spontaneum* is a very complex polyploid plant which possess approximately 26 types of chromosome number (2n = 40–128) [4]. In China, about 16 types have been reported with chromosome number ranging from 54 to 108, but only four ploidy clones (2n = 64, 72, 80, 96) appear to be distributed with high frequency [17,37–38]. However, the questions of how these ploidy clones evolved, their genetic relationships, and which ploidy clones have high breeding value for improving of sugarcane cultivar still remain unanswered. In this study, the analysis result of variable site analysis and haplotype diversity showed that decaploid and octaploid performed rich genetic variances. For the genetic relationship of four euploid populations of *S*. *spontaneum*, it was first illustrated according to rDNA ITS sequences. No obvious population differentiations appeared among four ploidy populations because of their small coefficient of gene differentiation and high gene flow value. This may be due to overlapping of their distribution regions, natural crossing with each other lead to high gene exchanging among populations.

Regarding the origin of *S*. *spontaneum* in china, Chen et al. [11] hypothesized that *S*. *spontaneum* might have originated from southern regions of Yunnan in China which has low altitude and latitude. They conjectured that it then spread to northwest regions of Yunnan with a higher altitude and latitude, then through Sichuan and Guizhou, and finally extended to other provinces such as Guangxi, Guangdong, Fujian, Jiangxi, and Zhejiang. Because octaploid clones are mainly distributed in possible origin regions such as Yunnan [17, 37–38], we inferred that octaploid clones might belong to a primitive chromosome type. According to chromosome number of nonaploid clone (2n = 72), we presumed that nonaploid clones may have arisen from a crossing of offspring between the octaploid clones (2n = 64) and decaploid clones (2n = 80) due to the overlap in their distribution regions. Because of 40 chromosomes from decaploid and 32 from octaploidy, the nonaploid should have a more close genetic relationship with decaploid than with octaploid. The genetic distance of three ploidy populations in this study is consistent with our assumption.

For Dodecaploid, it only distributed in Fujian provinces in China. Because its distribution region belongs to the extended regions of the evolution of *S*. *spontaneum*, we conjectured that dodecaploid clones may belong to evolutional types. Sreenivasan [39] hypothesized that it may originate from a triploid seedling from an octaploid, but the theory not be supported by our study. Actually, dodecaploid has a more close relationship with nonaploid rather than octaploid and decaploid, it means that dodecaploid may derived from nonaploid. But how they evolve still remains unknown, we presumed that the odd ploidy clone may produce a kind of six ploidy gamete containing 48 chromosomes, then crossing with each other form dodecaploid clone possessing 96 chromosomes. More research about the evolution of different ploidy of *S*. *spontaneum* should be carried out in future.
